# Mental Well-Being of Nursing Staff and Destructive Role of COVID-19 Fear and Perceived Stress

**DOI:** 10.3390/brainsci13071047

**Published:** 2023-07-09

**Authors:** Rima H. Binsaeed, Adriana Grigorescu, Ciprian Laurentiu Patru, Zahid Yousaf, Abdelmohsen A. Nassani, Larisa Patru (Grigorie)

**Affiliations:** 1 Department of Management, College of Business Administration, King Saud University, P.O. Box 71115, Riyadh 11587, Saudi Arabia; rbinsaeed@ksu.edu.sa (R.H.B.); nassani@ksu.edu.sa (A.A.N.); 2 Department of Public Management, Faculty of Public Administration, National University of Political Studies and Public Administration, Expozitiei Boulevard, 30A, 012104 Bucharest, Romania; 3 Academy of Romanian Scientists, Ilfov Street 3, 050094 Bucharest, Romania; 4 Department 8 Mother and Chid, University of Medicine and Pharmacy from Craiova, Petru Rares Street, 2, 200349 Craiova, Romania; ciprian.patru@umfcv.ro; 5 Higher Education Department, Government College of Management Sciences, Mansehra 21300, Pakistan; 6 Institute of National Economy, Romanian Academy, 13 Septembrie Street, 13, Sector 5, 050711 Bucharest, Romania; grigorie_larisa@yahoo.com

**Keywords:** COVID-19 fear, health crisis, perceived stress, mental well-being

## Abstract

Health crises across the globe bring dramatic changes to the lives of individuals and organizations. These crises have some psychological effects on society. The COVID-19 pandemic also caused some serious problems for individual and organizational life. Globally, the COVID-19 situation precipitated various economic and psychological issues that are far-reaching and exceptional. Health crises are increasing following the occurrence of COVID-19 due to its psychological effects on individuals worldwide. The current study highlighted the impact of COVID-19 fear on mental well-being (MWB). Most studies have examined the MWB of nursing staff and related their MWB to psychological factors. Few studies have considered the health crisis factors that are important in terms of bringing about variation in the MWB of nursing staff. Nursing staff MWB is impacted by various health crises (including COVID-19) at the global level and it has been ignored by researchers. In this study, a list of 1940 healthcare units with 6758 nursing staff was obtained. A total of 822 nurses were selected with the help of random sampling. The collected data were analyzed using correlation analysis, SPSS (statistical package for social sciences) version 23, and SEM. Thus, in this study we examined the effect of a health crisis (i.e., COVID-19) fear on the MWB of nurses. Moreover, we also examined the extent to which perceived stress (PS) influences the link between COVID-19 fear and MWB. The study’s findings confirmed that COVID-19 fear shown negative effect on MWB, while PS mediated the link between COVID-19 fear and MWB.

## 1. Introduction

The health crisis precipitated by the pandemic has posed various psychological issues and threats to all kinds of individuals as well as organizations, but the nursing professionals of both developed and developing economies have been the most badly affected [1,2]. Health crises around the globe have generated numerous economic and psychological issues that are far-reaching and exceptional [3,4]. Health crises have been increasing following the occurrence of COVID-19 due to its psychological effects on individuals worldwide [5]. These pandemic issues also affect the operational mechanisms of individuals and business organizations. The same is true for the healthcare sector, which is affected by a number of internal and external factors. The MWB (mental well-being) of nurses is affected by unpredictable crises. The healthcare sector is more susceptible to these crises than other sectors [1,6]. The MWB of nursing professionals is largely dependent on the prevailing conditions in the countries in which they work. Recently, decisions related to travel, social distancing, crowd restrictions, and community lockdowns have badly affected the working conditions and MWB of nurses [2,7]. However, the nature of, the exceptional conditions related to, and the fear of COVID-19 show that this crisis also has a major impact on MWB. Therefore, the aim of this research was to highlight the psychological effect of COVID-19. In this regard, we try to relate COVID-19 fear among nursing staff with their MWB.

The appearance of pandemic conditions exerts pressure on the healthcare sector to implement major health and innovation strategies in order to respond to the corresponding health crisis and improve the performance and MWB of nursing professionals [8]. There are multiple psychological and emotional factors that affect MWB during a health crisis. Among these psychological and emotional factors, perceived stress (PS) is an important factor that has a negative impact on the MWB of nursing professionals during a health crisis [9,10,11]. Existing studies have only documented how COVID-19 fear is a direct determinant of PS; therefore, this study considered the mediation of PS

Moreover, previous literature explained that COVID-19 negatively affects the MWB of nursing professionals [12,13]. COVID-19 fear plays a destructive role with respect to the MWB of nursing staff in the healthcare sector. Existing studies have documented various factors that play a mediating role in the link between COVID-19 fear and MWB (e.g., [14,15,16]). In this study, we argue that PS enhances the effect of COVID-19 fear on the MWB of nurses. Researchers in the relevant field have discussed the notion that stress is one of the significant determinants that increase the strain and mental and physical distraction of nursing professionals. Recently, nursing professionals, e.g., nurses, presented higher stress during COVID-19 [17]. Stress among nursing staff has increased due to the various mental and physical strains engendered by the COVID-19 pandemic [18,19], which reduce the MWB of nurses. A higher level of MWB among nursing staff becomes essential for allowing the healthcare sector to respond in a significant way during a pandemic situation [12]. 

This study reveals that the MWB of nurses can be damaged by COVID-19 fear and PS. The study in hand contributes to the existing literature on MWB in important ways. Moreover, the current research shows how COVID-19 fear plays a destructive role with respect to reducing the MWB of nurses. Furthermore, the PS among nursing staff due to COVID-19 also affects MWB in a negative way. 

## 2. Literature Review

### 2.1. COVID-19 Fear and MWB

MWB concerns satisfaction with life, positive and negative emotions, and a focus on meaning in life [20,21]. Cvenkel’s [22] study showed reduced concern in human resource management toward mental health and MWB and the importance of their enhancement in the framework of workspace well-being. In addition, happiness and MWB were studied, and Gray et al. [23] offer an overview of the existing literature. The MWB of nurses is mostly linked with the satisfaction of nursing staff. The MWB of nurses largely depends on the prevailing conditions in the countries they work in, which are considered the primary determinant of the MWB of nursing staff [24,25,26]. Stability in the economic and political environment strengthens the power of individuals regarding their spending decisions. With high levels of income, individuals can spend money on acquiring services [27]. On the other hand, worsening economic, political, and health crises badly affect individuals’ power and behavior regarding their spending on healthcare services [25]. In the last few years, the performance of the healthcare sector has declined due to the high levels of health crises across the globe [28,29]. Activity levels are improved according to one’s level of well-being at their respective office, and studies have demonstrated the importance of MWB to workers’ performance [30]. The COVID-19 pandemic has created psychological issues, which, in turn, change the behavior of individuals [31,32]. However, psychological aspect of the COVID-19 crises is fear among individuals around the globe. COVID-19 fear influences the behavior, motivations, and aspirations of individuals, which occasionally has destructive effects [33,34]. COVID-19 fear among nursing staff influences their activities, which badly affects their performance and MWB [25,28,35]. 

It is self-evident that nursing staff behavior plays an important role in and is considered one of the influencing factors of performance in the healthcare sector [36]. The fear of COVID-19 among nursing staff extensively detracts from their healthcare activities and MWB. Consequently, the nursing staff’s fear regarding COVID-19 badly impacts overall performance and MWB. Gorgenyi-Hegyes et al. [37] explored well-being during COVID-19 in Hungary, and their findings highlighted the importance of mental and emotional well-being in a workspace during a crisis.

**H1:**  *COVID-19 fear has negative effect on the MWB.*

### 2.2. COVID-19 Fear and PS

Social distancing and the shifting of duties induce mental disturbances among the nursing staff in healthcare departments [38,39]. Excessive admission of patients and the rescheduling of work exert pressures on nursing staff, leading to high levels of depression and stress at the workplace. Changes in duties and schedules and COVID-19 fear bring about psychological strain among nursing staff. COVID-19 fear increases mental strain and precipitates stress among nurses [40]. The psychological strain affects the work and family lives of nursing professionals. PS among nursing staff badly affects the working activities and reduces the MWB of the nurses [41,42]. COVID-19 fear increases dissatisfaction among nursing staff, which increases stress and depression [43]. Social distancing, working conditions, and restrictions during COVID-19 increase strain and the burden on nursing staff, leading to the development of stress [44]. PS can be significantly generated by pertinent influencing factors or be increased by an accurate fear, justified or not, amplified by public messages, and a lack of stress management within an organization or public perception [45,46].

**H2:**  *COVID-19 fear positively predicts PS.*

### 2.3. Mediating Role of PS

PS is related to psychological strain resulting from additional burdens and fatigue [16]. The emergence of the COVID-19 health crisis increased stress [40]. COVID-19 fear among nursing staff is a potential agent that reduces MWB [47]. With regard to maximizing the destructive role of COVID-19 fear, perceived stress plays a critical role in reducing the MWB of nurses [48]. PS can become a source of psychological strain due to the COVID-19 pandemic [16]. Recently, the healthcare sector, it has been largely acknowledged that COVID-19 fear leads to stress among nursing staff [49]. Researchers in the field of the healthcare sector have documented that PS is a significant predictor of the MWB of nurses [50,51]. Therefore, nursing staff perceive more stress due to health crisis pressures, which, in turn, affects their MWB. The view of the MWB of nurses has been extensively deliberated over, but only a few determinants and preconditions in relation to the psychological well-being of nursing staff have been considered. Organizational support and management implications can significantly influence the evolution of MWB in a workspace [52]. MWB has recently become a topic of research during the current health crisis; however, few studies have focused on the antecedents of MWB, particularly in the context of the healthcare sector.

**Figure 1:**
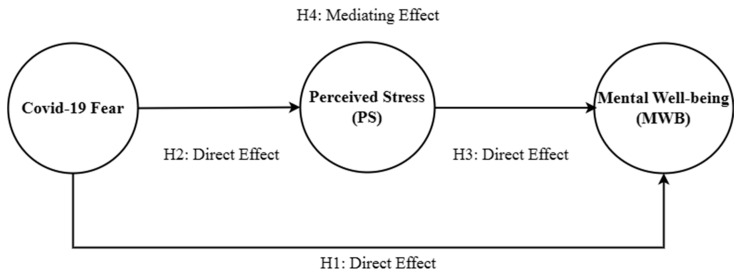
Theoretical Framework.

PS influences the link between COVID-19 fear and MWB. Study Hypothesis 1 explains the function of COVID-19 fear with respect to reducing MWB. On the other hand, we also hypothesize that COVID-19 fear increases the PS of nursing staff. Figure 1 presents the relationships among the study constructs.

**H3:**  *PS is negatively related to MWB of nurses.*

**H4:**  *PS mediates the connection between COVID-19 fear and the MWB of nurses.*

## 3. Methodology

The framework of the current study corresponds to a cross-sectional design. In the current study, we used questionnaire for data collection from the study respondents. The survey tool were pre-checked by four academia and seven field experts from different industry areas to make sure that items were valid. Based upon on pilot survey feedback, few adjustments were done in survey questions. The questionnaire was send through cover letter describing rationale of study and clearly stating that who has officially agreed to take part in study were providing information concerning confidentiality and data about critical variables would be use only for research purposes. A statistical correlation and PSL-AMOS was used to confirm the association between studies constructs. 

### 3.1. Context and Sample

The study population and sample frame consisted of the healthcare sector of Pakistan. Data were collected from nursing staff in the healthcare sector. To obtain information about the study sample, we approached the Ministry of Health of Pakistan. A list of 1940 healthcare units with 6758 nursing staff was received. A total of 822 nurses were selected with the help of random sampling. Selected nurses were approached with the help of a research assistant hired for the proper execution of research activities. Questionnaires were distributed to the selected sample to obtain responses relating to the study constructs. The questionnaire items were designed in both English and Urdu to make it easy for the participants to respond. During the four months of data collection we collected 625 responses. For the final analysis, we only included 582 responses.

### 3.2. Study Measures

The independent variable COVID-19 fear was measured based on the physical, mental, or psychological aspects of individuals. COVID-19 fear was measured using seven items adapted from existing studies, e.g., the study conducted by Ahorsu et al. [53], on the topic of COVID-19 fear that was relevant to the current research. 

The MWB of every individual was disturbed by the COVID-19 pandemic, which reduced their happiness and positive emotions and thus their MWB. The MWB of nurses was measured with 14 items adapted from Warwick–Edinburgh Mental well-being scale [54]. 

Finally, the mediating variable PS is based on the strain provoked by COVID-19 fear. PS was measured using seven items adapted from Zurlo et al. [55] and Lazarus and Folkman [56]. The measurement items are presented in Appendix A. The study questionnaire contained items measuring COVID-19 fear, PS, and nurses’ MWB. Table 1 contains information on the study constructs.

### 3.3. Statistical Techniques and Analysis

In order to test the study hypotheses, SEM (structural equation model) and SPSS (statistical package for social sciences) version 23 were used to perform the analysis. Hypotheses were tested with descriptive and inferential statistics at four levels model. First, the study constructs of the study was set up based on Cronbach’s alpha values. The results indicated that all constructs are reliable, as Cronbach’s alpha values were above the threshold value of 0.70. After the confirmation of reliability, data were entered into SPSS in order to statistically prove the study hypotheses. Moreover, we conducted descriptive and bivariate Pearson correlation analysis in SPSS based on the demographic information and responses from the study respondents. Correlation coefficients indicate the direction of an association between construct of the study. If the coefficient of correlation for two variables is greater than 1, this shows a strong and positive correlation and vice versa. In the current study, the value of correlations was greater than 1, thus confirming that there was a strong association between the study constructs.

## 4. Results

### 4.1. Reliability and Validity Analysis

Table 2 presents the findings of the reliability and validity analysis. The outcomes regarding the factor loading confirmed that the factor used actually measured what was intended. The AVE for each construct indicated good internal consistency of the constructs. Overall, the findings of the reliability and validity analysis confirmed the constructs’ reliability and discriminant and convergent validity.

Table 3 contains the results regarding the correlations between the study variables. The COVID-19 fear has a significant correlation with PS (0.25**) and MWB (0.17**). Furthermore, PS also has a significant correlation with MWB (0.23**). The findings presented in Table 3 confirmed the correlation between the study variables. On the basis of these correlation statistics, we tested the study hypotheses using further statistical analysis. 

### 4.2. Hypothesis Testing

In the current study, four hypotheses were formulated that explained the direct and indirect associations between study constructs. H1 highlighted the negative role of COVID-19 fear on MWB. This study’s hypotheses were tested using the SEM approach. Table 4 contains the statistics of path analysis, which are used to decide whether to accept or reject a study’s hypotheses. The direction of the first path explained the negative effect of COVID-19 fear for MWB. Path analysis confirmed the direct effect and significant degree of direction of each path. H1 of the study in hand determine the direct effect of COVID-19 fear on the MWB of nurses. The statistics generated for H1 confirmed the direct effect. Therefore, the coefficient (−0.17**) confirmed the acceptance of study H1. In line with the findings from the path analysis, we accept H1 of the current study. H2 explained the direction of the association between COVID-19 fear and PS. H2 was formulated to determine the direct link between COVID-19 fear and PS. The findings of the path analysis (shown in Table 4) confirmed that there was a direct link between COVID-19 fear and PS. The statistics, such as (0.25**), revealed that COVID-19 fear can positively predict the PS of nurses. The third path shows the direction of PS with respect to MWB. H3 also showed that PS negatively predicted the MWB of nurses. The findings depicted in Table 4 concern the path from PS to MWB. The coefficient (−0.21**) obtained statistically proved the negative effect of PS on the MWB of nurses. The results presented in Table 4 reveal that PS predicted MWB; therefore, we accept H3.

**Figure 2:**
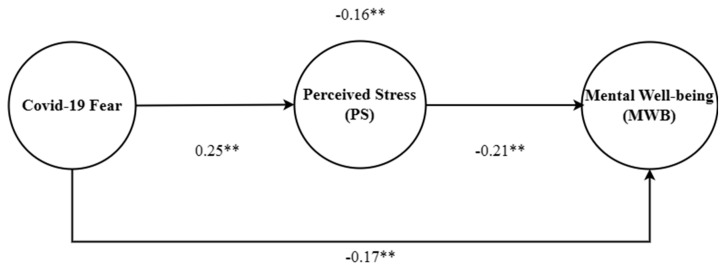
Results of Path Analysis for Mediation Model (** *p* < 0.01, two-tailed).

Finally, H4 was formulated to determine whether PS mediates the link between COVID-19 fear and MWB. Table 5 contains the statistics regarding the mediation of PS. On the basis of the statistics obtained concerning the indirect effect of PS, confirmed the mediating effect of PS. The findings suggested that COVID-19 crises increase the PS which in turn reduces the MWB. The coefficient of the indirect effect (−0.16**) confirmed the mediating effect between the COVID-19 fear and MWB relationship. Based on the outcome obtained regarding the indirect effect of PS, we accept H4. In summary, the four hypotheses have been accepted based on the statistics obtained through the path analysis techniques of SEM. The statistics support the study’s arguments regarding the negative role of COVID-19 fear with respect to MWB. Furthermore, the empirical findings also support the intervening role of PS. Figure 2 presents the findings of path analysis for the mediation model.

## 5. Discussion

This study provides valuable contributions and in-depth knowledge about how COVID-19 fear affects the MWB of nursing staff. The emergence of a health crisis in the last few years has posed serious challenges for all kinds of organizations with respect to their survival, performance, and health. The same is true for the healthcare sector. In this study, we formulated four hypotheses. First, COVID-19 fear is one of the destructive factors that have emerged from the corresponding health crisis. COVID-19 fear affects the behavior, thinking, and motivation of individuals across the globe and is considered one of the critical factors reducing the mental and physical health of nurses. Previous findings support the notion that COVID-19 fear predicts MWB [25,27].

Our second hypothesis supports the notion that PS negatively predicts the MWB of nursing staff working in the healthcare sector of Pakistan. Through PS due to COVID-19, fear reduces the MWB of nursing staff. It is impossible for nursing staff to properly perform their activities under stressful conditions. PS reduces the MWB of nursing staff due to COVID-19 fear within the healthcare sector. H3 of this study examined that how MWB is affected during COVID-19 crises in the presences of PS. In this study we proposed that PS of the nurses increases with the level of health crises which provide the foundation for the disturbance of MWB. The findings of the study confirmed the intervening role of PS. The COVID-19 pandemic led to the imposition of various kinds of restrictions such as social distancing, lockdowns, and duty rescheduling, leading to psychological strain among individuals, which increases stress and thus badly affects MWB. The statistics of indirect effect confirmed the acceptance of H3.

In accordance with the current study findings PS plays an intervening role. Therefore, we formulated H4 that explained the PS mediating role on the relation between COVID-19 fear and MWB. We suggested that the emergence of COVID-19 health crisis increased stress [40]. Moreover, the COVID-19 fear among nursing staff is a potential agent that reduces MWB. With regard to maximizing the COVID-19 fear disruptive role, PS significantly affect in terms of reducing the MWB of nurses [47,48]. The current study argued that PS can become a source of psychological strain due to the COVID-19 pandemic. Researchers in the field of the healthcare sector have documented that PS is a significant predictor of the MWB of nurses [50,51]. Therefore, nursing staff perceive more stress due to health crisis pressures, which, in turn, affects their MWB. The nursing stuff is facing the stress increase by the COVID-19 fear, which negatively affects their MWB. PS plays a destructive role for MWB and increases the COVID-19 fear devastating role with respect to MWB. 

### 5.1. Theoretical Contribution

The existing literature gain the contribution of the current study on health crises, stress, and mental health. The major contribution of the current study is the formulation of a model that includes COVID-19 fear as a destructive factor with respect to the MWB of nursing staff. Firstly, we developed a model containing the health crisis factor, particularly in terms of the healthcare sector, in order to highlight the direct effect of COVID-19 fear and the mediating effect of PS with regard to increasing the destructive influence of COVID-19 fear on MWB.

Secondly, the contribution to the existing literature consists in finding an explanations for the role of PS in reducing the MWB of nursing staff. PS is considered a critical factor that plays a destructive role in MWB [48,50,51]. Limited literature is available on the destructive role of PS with respect to its combination with COVID-19 fear. Therefore, we considered this research aspect and filled this gap, focusing on PS as a potential mediator in the relationship of COVID-19 fear and the MWB.

### 5.2. Practical Implications

The outcome of the research offers valuable suggestions for management in practice. First, the study findings revealed that the healthcare sector can improve the MWB of nursing staff during a health crisis by reducing stress. By doing so, adequate MWB of nursing staff is possible when these healthcare sectors opportunely react to the consequences of COVID-19 fear. Second, this study shows that providing the proper facilities to nursing staff during health crises reduces their stress and improves their MWB. Therefore, through the proper management of health crises, the healthcare sector can easily minimize the effect of COVID-19 fear on nursing professionals and strengthen their MWB. 

## 6. Conclusions

Health crises such as COVID-19 have various psychological effects on individuals worldwide. The study purpose was to examine the destructive influence of COVID-19 fear on MWB. Existing studies ignore the psychological factors such as COVID-19 fear that are important to bring about variation in the MWB of nursing staff. Therefore, we examined the effect of a health crisis, i.e., COVID-19 fear, on MWB. Moreover, we also examined the extent to which PS intervenes in the relationship between COVID-19 fear and MWB. We have formulated that COVID-19 fear is one of the destructive factors of MWB. COVID-19 fear affects the behavior and psychological state of individuals, which badly affects their decisions regarding nursing activities. The most destructive aspect of MWB is PS under the COVID-19 pandemic. The results confirmed that COVID-19 fear reduces MWB. Furthermore, the statistical findings suggested that PS further enhances the negative effect of COVID-19 fear and mediates the relationship between COVID-19 fear and MWB. This study’s findings confirmed that COVID-19 fear has a destructive influence on MWB, while PS mediates the connection between COVID-19 fear and MWB. 

## Figures and Tables

**Table 1:** Measurement scale.

Construct	Method	Authors	Number of Items
COVID-19 Fear	Adapted	[53]	7 items
MWB	Adapted	[54]	20 items
PS	Adapted	[55,56]	7 items

Source: authors’ computation.

**Table 2:** Reliability and validity of constructs.

	Items	Cronbach’s Alpha	Factor Loading	Composite Reliability	AVE
COVID-19 Fear	7	0.79	0.73–0.91	0.82	0.68
MWB	20	0.76	0.70–0.88	0.80	0.71
PS	7	0.81	0.76–0.90	0.84	0.73

Source: authors’ computation.

**Table 3:** Correlation.

Constructs	Mean	SD	Gender	Age	Experience	Education	COVID-19 Fear	PS	MWB
Gender	0.87	0.84	1						
Respondent age	34	---	0.09	1					
Work experience	2.6	0.84	0.08	0.03	1				
Education level	2.4	0.91	0.06	0.05	0.04	1			
COVID-19 fear	3.8	0.93	0.09	0.12	0.08	0.07	1		
PS	3.5	0.91	0.05	0.09	0.04	0.05	0.32 **	1	
MWB	3.6	0.90	0.08	0.03	0.04	0.09	−0.25 **	−0.23 **	1

Source: authors’ computation. Note: ** *p* < 0.01, two-tailed: SD (Standard Deviation); PS (perceived stress); and MWB (mental well-being).

**Table 4:** Path analysis.

Paths	Estimates	Standard Error	C.R (t-Value)
COVID-19 fear → MWB	−0.17	0.063	2.698 **
COVID-19 fear → PS	0.25	0.057	4.385 **
PS → MWB	−0.21	0.059	3.559 **

Note: ** *p* < 0.01, two-tailed: *CR (critical ratio). Source: authors’ computation.*

**Table 5:** Results concerning the indirect effect of PS.

Specification	Estimate	LL	UL
*Standardized direct impact*			
COVID-19 fear → MWB	−0.17	−0.05	0.27
COVID-19 fear → PS	0.25 **	0.39	0.58
PS → MWB	−0.21 **	0.19	0.50
*Standardized indirect effects*			
COVID-19 fear → PS → MWB	−0.16 **	0.07	0.27

Note: ** *p* < 0.01, two-tailed: LL (lower limit) and UL (upper limit). Source: authors’ computation.

## Data Availability

Data available on request.

## Appendix A. Study measures

*COVID-19 Fear*

1. I am afraid of Coronavirus-19 contamination
2. I fill uncomfortable thinking on the coronavirus-19 hazard
3. When I am thinking about coronavirus-19 my body lose control
4. I become anxious and nervous when I am watching news about coronavirus-19
5. I am afraid of high risk to die by coronavirus-19 disease
6. I am worried and my slip is affected by the fear of coronavirus-19 contamination
7. When I think about getting coronavirus-19 I am devastated

Perceived Stress

How do you perceive during COVID-19 about the….

1. risk of contagion
2. condition of social isolation?
3. relationships with your relatives
4. relationships with your colleagues
5. relationships with your health care professionals
6. performance experience
7. your love and sexual life and social isolation

*Mental well-being*

I have been…

1. Felling optimistic about the future
2. Felling useful
3. Feeling relaxed
4. Interested in other people
5. Energy to spare
6. Dealing with problem well
7. Thinking clearly
8. Feeling good about myself
9. Feeling close to other people
10. Feeling confident
11. Able to make up my own mind about things
12. Feeling loved
13. Interested in new things
14. Felling cheerful
